# Supplementary material for: Regulatory protein SrpA controls phage infection and core cellular processes in Pseudomonas aeruginosa
Source: Nat Commun. 2018 May 10;9:1846. doi: 10.1038/s41467-018-04232-6 (PMC5945682; doi:10.1038/s41467-018-04232-6)
Supplement: Supplementary file 1 — Supplementary Information [file 41467_2018_4232_MOESM1_ESM.pdf]

Regulatory protein SrpA controls phage infection and core cellular processes in *Pseudomonas aeruginosa*

You et al

Supplementary Figure 1

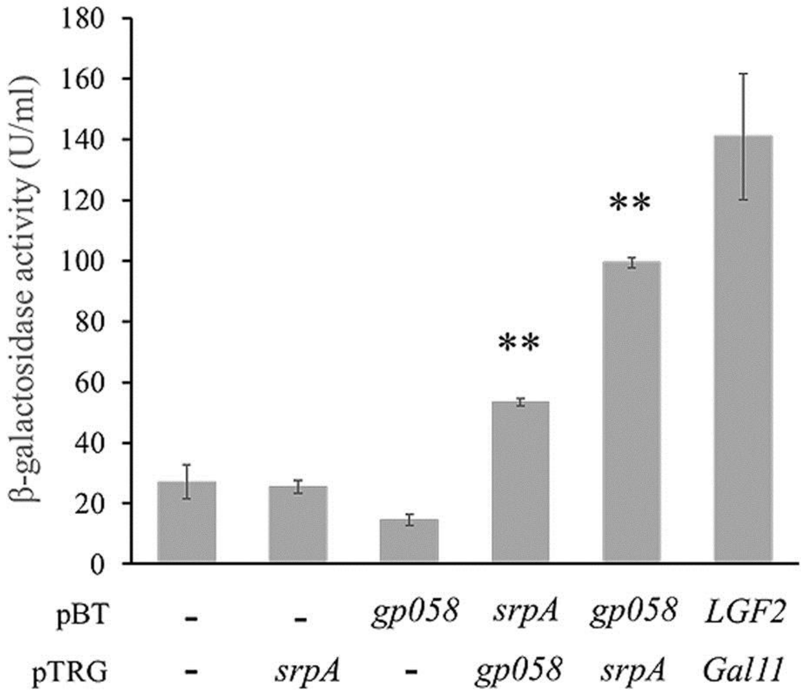

Supplementary Figure 1. Bacterial two-hybrid analysis. Negative controls: pBT+pTRG, pBT +pTRG-*srpA*, and pBT-*gp058*+pTRG. **: P<0.01, all three negative controls show significant differences from the test groups of pBT-*srpA* +pTRG-*gp058* and pBT-*gp058*+pTRG-*srpA*, respectively. Positive control: pBT-*LGF2* +pTRG-*Gal11*. The experiments were independently replicated three times. One-way ANOVA was used to examine the mean differences between the data groups. **: P<0.01. Error bars show standard deviations.

Supplementary Figure 2

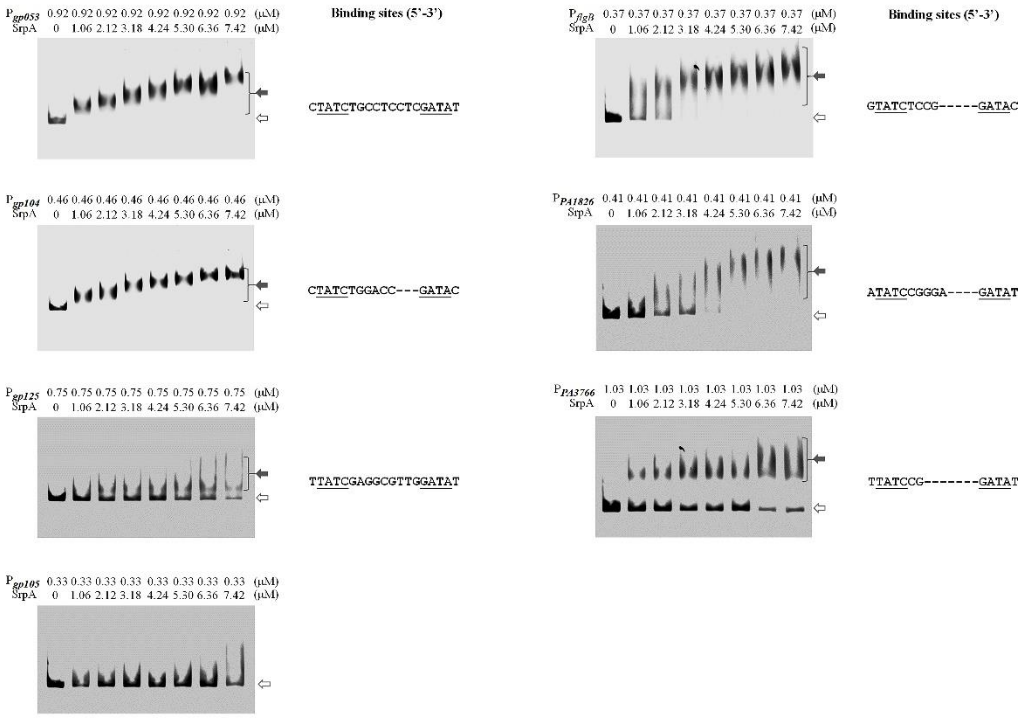

Supplementary Figure 2. Verification of the SrpA binding sites. Left panel: gel-shifting assay of the promoter regions of three phage K5 genes. Right panel: gel-shifting assay of the promoter regions of three bacterial genes. The *gp105* promoter without a SrpA binding site was used as a negative control. Grey arrows represent DNA fragments bound with the SrpA protein. Blank arrows represent free DNA fragments. The palindromic sequences of each binding site were underlined.

Supplementary Figure 3

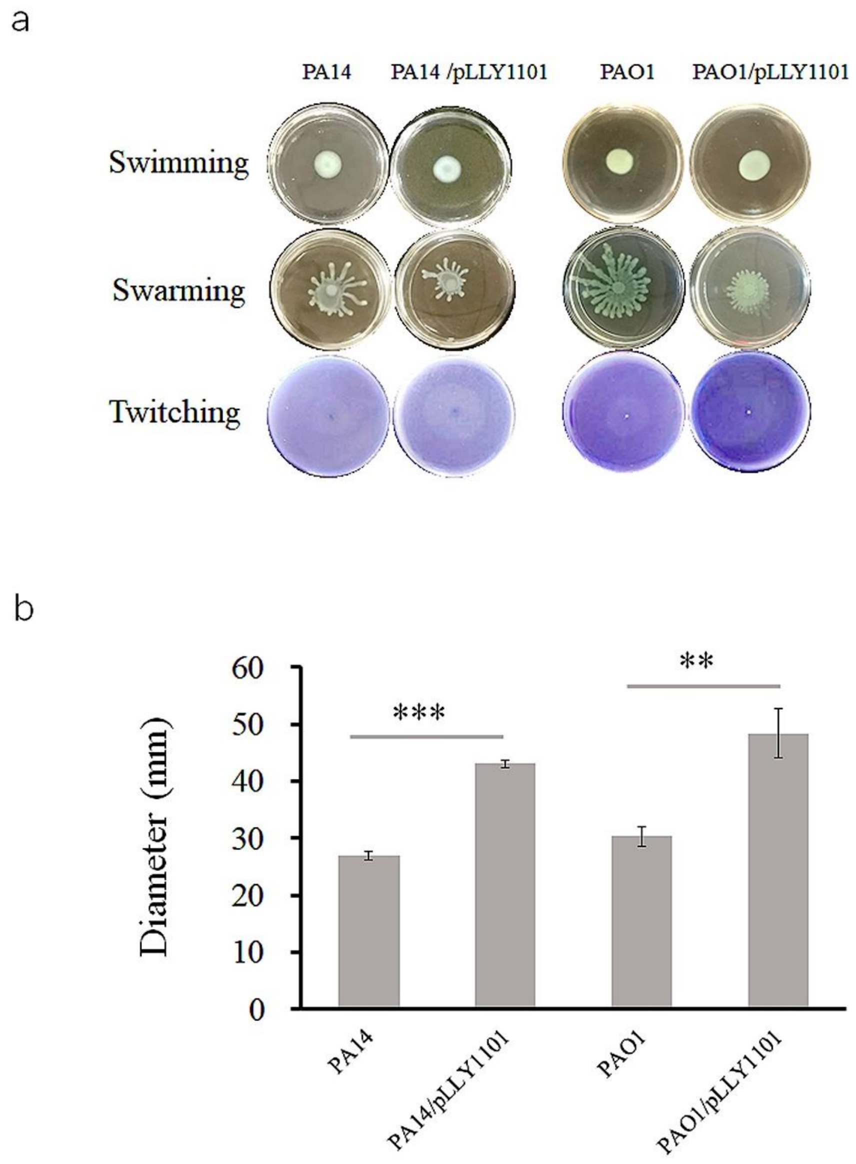

Supplementary Figure 3. Effects of SrpA on cell motility in PAO1 and PA14. (a) The upper panel represents swimming assay. The mid-panel represents swarming assay. The lower panel and (b) represents twitching motility assay. SrpA was overexpressed in the strains PAO1 and PA14 (Supplementary Table 2). The plasmid pLLY1101 carries the *srpA* gene. The experiments were independently replicated three times. One-way ANOVA was used to examine the mean differences between the data groups. **: P<0.01. ***: P<0.001. Error bars show standard deviations.

Supplementary Figure 4

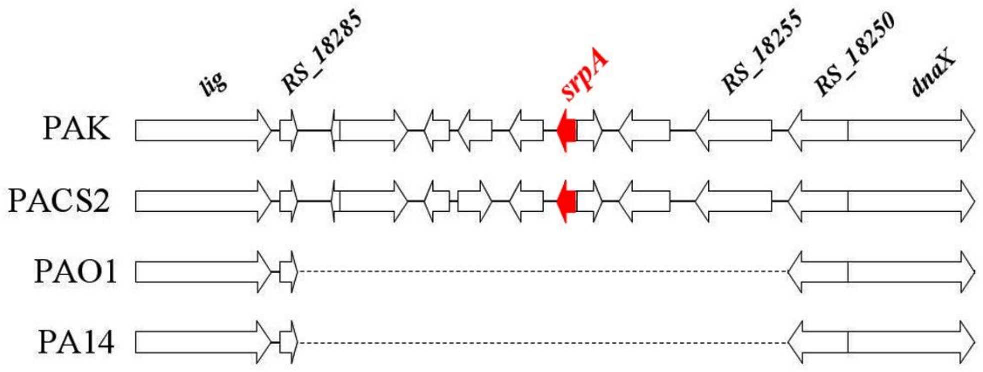

Supplementary Figure 4. The genomic context of the *srpA* gene. Both PAK and PACS2 have a 7.2 kb fragment carrying the *srpA* gene inserted between two genetic loci *dnaX* and *lig*. *dnxX*: encoding a putative DNA polymerase subunits gamma/tau. *lig*: encoding a putative DNA ligase. *Y880_RS18250*: encoding a putative amino acid ABC transporter substrate-binding protein. *Y880_RS18255*: encoding a putative integrase. The remaining CDSs encode hypothetical proteins.

Supplementary Figure 5

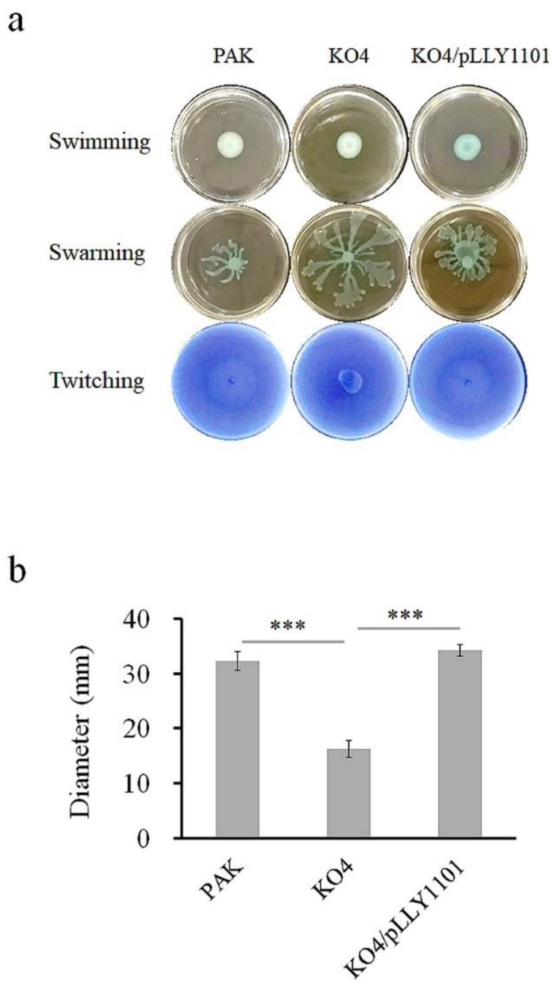

Supplementary Figure 5. Cell motility assay of the *srpA* gene deletion mutant. (a) The upper panel represents swimming assay. The mid-panel represents swarming assay. The lower panel and (b) represents twitching motility assay. KO4 was the *srpA* gene deletion mutant in PAK (Supplementary Table 2). The plasmid pLLY1101 carries the *srpA* gene. The experiments were independently replicated three times. One-way ANOVA was used to examine the mean differences between the data groups. ***: P<0.001. Error bars show standard deviations.

Supplementary Figure 6

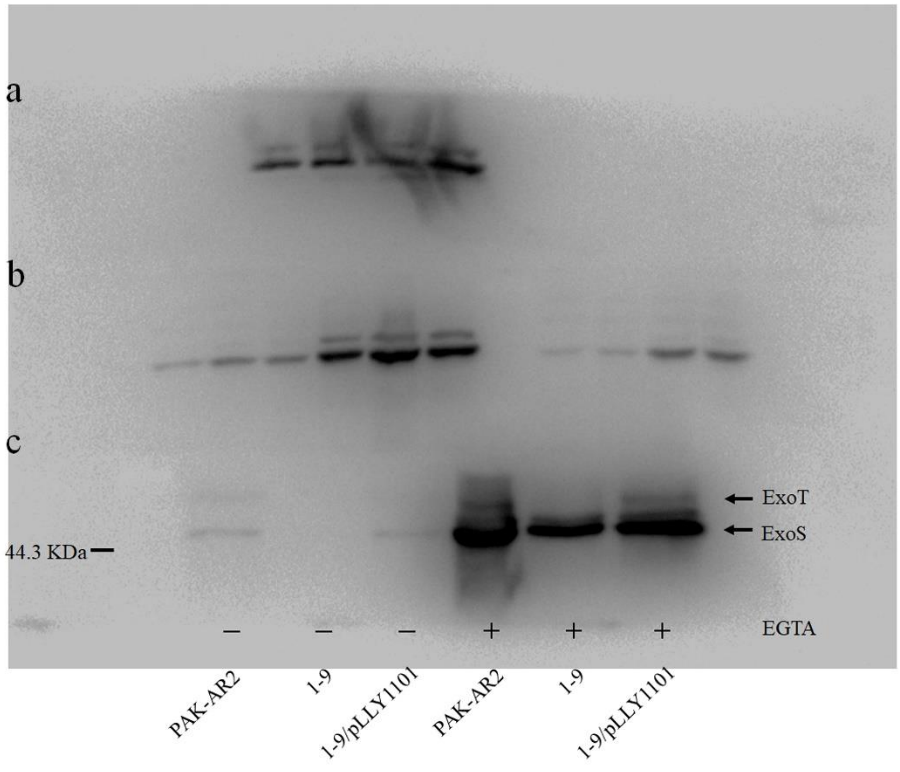

Supplementary Figure 6. The full blots of the detection of secreted ExoS and ExoT in bacterial culture supernatants by Western blot. The upper panel (a) and the middle panel (b) represent the experiments not related with our test, and the lower panel (c) represents the original image for our Western blot analysis*. 1-9: a *srpA* derivative of PAK-AR2. pLLY1101: a plasmid carrying the *srpA* gene (Supplementary table 2). Protein Molecular Weight Marker (Low) is used in the SDS-PAGE analysis, and it is from TAKARA BIOTECHNOLOGY (DALIAN) CO., LTD. (Code No. 3450).

*Three membranes from three different experiments were put together and exposed to the same piece of X-ray film simultaneously.

Supplementary Table 1. SrpA DNA binding sites upstream of bacteria or phage genes

Gene ID	Gene name	Start (bp)	End (bp)	log2 ratio (1-9A/PAK-AR2)	Binding sequence (5'-3')	Function
PA1077	*flgB*	1164061	1164073	1.3261	ATATCGACGATAT	flagellar basal-body rod protein FlgB
PA3115	*fimV*	3498367	3498383	0.3672	CTATCTGTAAAAGATAC	type IV pilus
PA2522	*czcC*	2843385	2843402	0.0202	ATATCGCCGGCGAGATAT	outer membrane protein precursor CzcC
PA3397	*fprA*	3803456	3803474	0.4781	ATATCCATATTCTGGATAA	cofactor biosynthetic process
PA4086	*cupB1*	4570040	4570051	0.1559	GTATCCCGATAT	probable fimbrial subunit CupB1
PA2760	*oprQ*	3119794	3119812	0.2843	TTATCGATAAGGTTGATAT	transport
PA0688	*lapA*	747052	747065	0.4781	GTATCGGCGGATAA	low-molecular-weight alkaline phosphatase A, LapA
PA2523	*czcR*	2843385	2843402	0.4294	ATATCTCGCCGGCGATAT	Two-component system
PA0873	*phhR*	953460	953469	0.3633	TTATCGATAC	transcriptional regulator PhhR
PA3763	*purL*	4219653	4219667	0.3078	CTATCGCCGGGATAC	phosphoribosylformylglycinamidine synthase
PA2512	*antA*	2829231	2829242	0.4075	CTATCCGGATAG	anthranilate dioxygenase large subunit
PA2511	*antR*	2829231	2829242	0.0805	CTATCCGGATAG	regulation of transcription
PA4277	*tufb*	4785575	4785592	-0.3597	TTATCCAGGGGCAGATAT	Translation elongation factor
PA1898	*qscR*	2069014	2069031	-0.4053	GTATCTGCGGAGAGATAG	quorum-sensing control repressor
PA1249	*apra*	1355573	1355587	-0.3333	TTATCCATCCGATAA	alkaline metalloproteinase precursor
PA4857	*tspR*	5455097	5455110	-0.0577	GTATCGGCGGATAA	protein secretion by the type III secretion system
PA5206	*argE*	5861656	5861674	-0.0189	TTATCCACTGCCGCGATAG	acetylornithine deacetylase
PA2493	*mexE*	2808639	2808657	-0.6738	GTATCACTGTTCGTGATAA	RND multidrug efflux membrane fusion protein MexE precursor
PA1727	*mucR*	1870802	1870816	-0.6088	ATATCAGGTTGATAT	Biofilm formation
PA3540	*algD*	3962465	3962474	-0.5002	CTATCGATAG	GDP-mannose 6-dehydrogenase AlgD
PA4581	*rtcR*	5130775	5130790	-0.7211	TTATCTTGAAGGATAA	transcriptional regulator RtcR
PA4267	*rpsG*	4771726	4771741	-0.7338	TTATCTGAGTCGATAA	30S ribosomal protein S7
PA4268	*rpsl*	4772247	4772258	-0.6371	CTATCCCGATAG	30S ribosomal protein S12
PA0293	*aguB*	330981	330991	-0.5280	ATATCCGATAA	N-carbamoylputrescine amidohydrolase
PA3745	*rpsP*	4306340	4306351	-1.1256	ATATCGAGATAA	30S ribosomal protein S16
PA0942		1030680	1030694	0.6289	GTATCGATACGATAC	probable transcriptional regulator
PA1826		1985940	1985954	1.2293	ATATCCGGGAGATAT	probable transcriptional regulator
PA2121		2333892	2333910	1.3992	TTATCAGGGTAATGGATAA	probable transcriptional regulator
PA0233		262426	262439	0.2591	TTATCCGCCGATAC	probable transcriptional regulator
PA3398		3803456	3803474	0.4352	TTATCCAGAATATGGATAT	probable transcriptional regulator
PA0179		204644	204653	-0.3672	GTATCGATAG	probable two-component response regulator
PA4070		4548861	4548870	-0.3667	ATATCGATAA	probable transcriptional regulator
PA4989		5606022	5606040	-0.4570	CTATCTTTATTGGAGATAG	probable transcriptional regulator
PA5157		5804947	5804958	-0.9291	ATATCTTGATAA	probable transcriptional regulator

PA0985	*pyos5*	1068020	1068032	-	TTATCAGAGATAC	pyocin S5
PA1094	*fliD*	1185009	1185023	-	CTATCGAAGGGATAG	flagellar capping protein FliD
PA1366		1479909	1479922	-	GTATCTCTCGATAG	hypothetical protein
PA0015		17339	17357	0.3254	CTATCGCGAACGGCGATAG	hypothetical protein
PA0042		54636	54648	0.1219	ATATCGGCGATAC	hypothetical protein
PA0136		154009	154023	-0.4878	ATATCTTGTGGATAA	probable ATP-binding component of ABC transporter
PA0941		1030680	1030694	0.4193	GTATCGTATCGATAC	hypothetical protein
PA0986		1068020	1068032	-0.5108	GTATCTCTGATAA	conserved hypothetical protein
PA1227		1329176	1329192	-0.5029	ATATCGCCGGGCGATAG	hypothetical protein
PA1362		1474154	1474168	0.6206	TTATCCGCCGGATAC	hypothetical protein
PA1415		1539728	1539744	-0.9615	CTATCCCAGAAGGATAG	hypothetical protein
PA1728		1870802	1870816	-0.0949	ATATCAACCTGATAT	hypothetical protein
PA1827		1985940	1985954	0.8780	ATATCTCCCGGATAT	probable short-chain dehydrogenase
PA1874		2036251	2036265	0.5110	TTATCAATTAGATAG	hypothetical protein
PA1873		2036251	2036265	-0.0298	CTATCTAATTGATAA	hypothetical protein
PA1897		2069014	2069031	0.5559	CTATCTCTCCGCAGATAC	hypothetical protein
PA2122		2333892	2333910	0.2662	TTATCCATTACCCTGATAA	hypothetical protein
PA2422		2705500	2705513	0.6882	GTATCGGCGGATAC	hypothetical protein
PA2573		2910642	2910657	0.7803	CTATCCTTAGCGATAT	probable chemotaxis transducer
PA2759		3119794	3119812	1.9617	ATATCAACCTTATCGATAA	hypothetical protein
PA2784		3141627	3141643	0.4252	TTATCGTTTTACGATAA	hypothetical protein
PA2868		3221098	3221112	0.2626	CTATCGCCCGGATAG	hypothetical protein
PA3520		3937875	3937884	-1.2753	TTATCGATAA	hypothetical protein
PA3764		4219653	4219667	-0.0716	GTATCCCGGCGATAG	hypothetical protein
PA3765		4221857	4221868	-0.3356	TTATCCGGATAA	hypothetical protein
PA3766		4223466	4223477	-1.0299	TTATCCGGATAT	probable aromatic amino acid transporter
PA3844		4306340	4306351	0.2028	TTATCTCGATAT	hypothetical protein
PA4087		4570040	4570051	1.3922	ATATCGGGATAC	hypothetical protein
PA4990		5606022	5606040	-0.0456	CTATCTCCAATAAAGATAG	SMR multidrug efflux transporter
PA5567		6261762	6261775	-0.3929	CTATCCGGAGATAT	conserved hypothetical protein
PA3230		3618607	3618620	0.1385	GTATCGGCGGATAA	conserved hypothetical protein
PA3284		3677316	3677332	0.0873	ATATCGCTGCGCGATAT	hypothetical protein
gp001 or *gp002*		303	312	-	CTATCGATAG	hypothetical proteins
gp055		28376	28385	-	TTATCGATAA	hypothetical protein
gp074		42415	42424	-	CTATCGATAC	hypothetical protein
gp177 or *gp178*		92875	92884	-	CTATCGATAG	hypothetical proteins
gp166		87558	87568	-	GTATCTGATAA	hypothetical protein
gp015		7268	7279	-	TTATCCTGATAA	hypothetical protein
gp140		77814	77827	-	CTATCCCTCGATAA	hypothetical protein

gp131	75323	75337	-	CTATCTTATAGATAA	hypothetical protein
gp147	81598	81612	-	GTATCCGTCTGATAT	hypothetical protein
gp009	5037	5052	-	GTATCCAGAGAGATAA	hypothetical protein
gp074	42613	42628	-	TTATCGAAAACGATAC	hypothetical protein
gp104	58950	58965	-	CTATCTGGACCGATAC	DNA polymerase I
gp053	27055	27073	-	CTATCTGCCTCCTCGATAT	DNA methyltransferase
gp058	31024	31042	-	GTATCTCCAAATCGGATAT	RNA polymerase
gp125	70698	70716	-	TTATCTCCAAATCGGATAT	hypothetical protein

a: No expression data is available for PA0985, PA1094, and PA1366 in the transcriptome analysis.

b: Annotation is performed using strain PAO1 as the reference.

c: Phage genes are named with prefix '*gp*'.

d: The SrpA binding sites are located at the upstream regions between two divergent genes pairs, *gp001* and *gp002* and *gp177* and *gp178*, respectively.

e: Two SrpA binding sites are found located at the upstream region of the *gp074* gene.

f: No expression test is performed for phage genes in the transcriptome analysis.

Supplementary Table 2. Bacterial strains and plasmids used in this study

Strain/ Plasmid /Phage/Nematode	Description	Source (Reference)
P. aeruginosa		
PAK	Wild type *Pseudomonas aeruginosa* strain	[1]
PAK-AR2	PAK deleted of the *purEK* gene, Sp^rSm^r	[2]
1-9	PAK-AR2 with Tn5G transposon inserted at *sprA*, Gm^r	This study
C11-1	PAK with Tn5G transposon inserted at *sprA*, Gm^r	[3]
KO4	PAK with *sprA* gene deletion	This study
PAO1	Wild type *P. aeruginosa* strain	[4]
PA14	Wild type *P. aeruginosa* strain	[5]
E. coli		
DH5α	*hsdR recA lacZYAF80 lacZDM15*	BRL
OP50	Uracil auxotroph	[6]
RS	BacterioMatch two-hybrid system reporter strain, Km^r	Stratagene
M15	Overexpression of *lacI*, Km^r	Qiagen
**Plasmid**		
pEx18Gm	Gene replacement vector, Gm^r *oriT*$^+$ *sacB*$^+$	[7]
pDN19lacΩ	Promoterless *lacZ* fusion vector, Sp^r Sm^r Tc^r	[8]
pUCP18	Broad host range shuttle vector, Ap^r	[9]
pUCP20	*Escherichia–Pseudomonas* shuttle vector; Ap^r	[10]
pUCP20-P_{higB}::*gfp*	*higA* promoter of PA14 fused to promoterless *gfp* on pUCP20; Ap^r	[11]
pBT	Bait vector encoding phage cI protein, Chl^r	Stratagene
pTRG	Target vector encoding RNAP α-subunit protein, Tc^r	Stratagene
pLLY1101	*srpA* gene driven by its native promoter in pUCP18, Ap^r	This study
pQE30	Fusion vector for N-terminal His tag, Ap^r	Qiagen
pYJJ1501	*his-srpA* fusion in pQE30 vector, Ap^r	This study
pYJJ1605	*gp058* gene with promoter region cloned in pUCP20, Ap^r	This study
pYJJ1606	*gp104* gene promoter cloned in pDN19lacΩ, Sp^r Sm^r Tc^r	This study
pYJJ1607	*gp105* gene promoter cloned in pDN19lacΩ, Sp^r Sm^r Tc^r	This study
pYJJ1610	*gp058* gene promoter cloned in pDN19lacΩ, Sp^r Sm^r Tc^r	This study
pQY1302	*gp056* gene promoter cloned in pDN19lacΩ, Sp^r Sm^r Tc^r	This study
pQY1303	*gp071* gene promoter cloned in pDN19lacΩ, Sp^r Sm^r Tc^r	This study
pQY1304	*gp128* gene promoter cloned in pDN19lacΩ, Sp^r Sm^r Tc^r	This study
pYJJ1612	*gp058* gene cloned in pBT, Chl^r	This study
pYJJ1613	*gp058* gene cloned in pTRG, Tc^r	This study
pYJJ1614	*srpA* gene cloned in pBT, Chl^r	This study
pYJJ1615	*srpA* gene cloned in pTRG, Tc^r	This study
pYJJ1606	*gp104* gene cloned in pDN19lacΩ, Sp^r Sm^r Tc^r	This study
pXJ1701	*gp058* gene driven by the P_{lac} promoter in pUCP20, Ap^r	This study
pHZW1701	1.9 kb DNA fragment in pEx18Gm for knocking out the *srpA* gene, Gm^r	This study
pMD19 (Simple)	A linearized T-vector for cloning promoter fragments, Ap^r	TaKaRa

pSL1701	*gp058* gene promoter cloned in pMD19 (Simple), Ap^r	This study
pSL1702	*gp104* gene promoter cloned in pMD19 (Simple), Ap^r	This study
pSL1703	*gp105* gene promoter cloned in pMD19 (Simple), Ap^r	This study
pSL1704	*PA1077* gene promoter cloned in pMD19 (Simple), Ap^r	This study
pSL1705	*PA1826* gene promoter cloned in pMD19 (Simple), Ap^r	This study
pSL1706	*PA3766* gene promoter cloned in pMD19 (Simple), Ap^r	This study
pSL1707	*gp053* gene promoter cloned in pMD19 (Simple), Ap^r	This study
pSL1708	*gp125* gene promoter cloned in pMD19 (Simple), Ap^r	This study
**Phage**		
K5	*Pseudomonas* phage	[12]
**Nematode**		
N2	Wild type *Caenorhabditis elegans* strain (ancestral)	[6]

Supplementary Table 3. Primers used in this study

Primers	Sequence (5'-3')	Target genes
OTn1	GATCCTGGAAAACGGGAAAG	Identification of Tn5G insertion sites with inverse PCR
OTn2	CCATCTCATCAGAGGGTAGT	
1-9-2F	CGCAAGCTTCAGGGGTGTCAAGAAAGATAAACCGC	Cloning *srpA* into pUCP18
1-9-2R	CGCGGATCCGGTCATCTCCATCTTTTTGCTGGGTCG	
58-F	GTTGGTTTCTTCCCCGAGG	qPCR primers, coding region of gene *gp058*
58-R	ATTCGTCTGCCTTCCATCGC	
S1	AGCCTGCTCTCTCCGTTC	DNA probe synthesis for southern blot
S2	TCTCAAGAGTGCTGTCCC	
DNAI-qF	CTTCCGTAAGCATTTGGC	qPCR primers, coding region of gene *gp104*
DNAI-qR	CAATGCCATACAGCGGTG	
DNAII-qF	CTGACCAAACAGTGGGAAGC	qPCR primers, coding region of gene *gp105*
DNAII-qR	TGAGCCGAACGGGTAGGA	
RNA-lacZ-F	CGGAATTCATCCAGCAAACAACCCTA	The promoter region of gene *gp058* for construction of transcriptional reporter
RNA-lacZ-R	CGGGATCCCCGTGTTACGTTGTCGGG	
DNAI-lacZ-F	CGGAATTCCAGCGGAGACAACCTAAA	The promoter region of gene *gp104* for construction of transcriptional reporter
DNAI-lacZ-R	CGGGATCCCATTTCCTTTCCTCCTGT	
DNAII-lacZ-F	CGGAATTCTTACACTGCCAGCCCCTA	The promoter region of gene *gp105* for construction of transcriptional reporter
DNAII-lacZ-R	CGGGATCCTCACACAGTTTTACATGGAC	
pbp-lacZ-F	CCGGAATTCACCTAGAACAGGTGCAGTATAAGC	The promoter region of gene *gp071* for construction of transcriptional reporter
pbp-lacZ-R	CGCGGATCCGACAGAAAGGCGTCTAAAATCGTC	
rnr-lacZ-F	CCGGAATTCACTTTTTCTACCTACGTCGTCACA	The promoter region of gene *gp128* for construction of transcriptional reporter
rnr-lacZ-R	CGCGGATCCTCTTCTAGTTGCTGGTTTGCTAGT	
cap-lacZ-F	CCGGAATTCGCAAAAGATCATCAGCGTCTTAG	The promoter region of gene *gp105* for construction of transcriptional reporter
cap-lacZ-R	CGCGGATCCGTTTCGATCTCTTCGAACTGGACA	
RNA-H-F1	CGGGATCCGTGGAGGCTCTAGACGGA	Cloning *gp058* into pTRG
RNA-H-F2	TCCCCCGGGGAGTGGAGGCTCTAGACGGA	Cloning *gp058* into pBT
RNA-H-R	CCCTCGAGTTATTGCCTCCGATGGATT	
srpA-H-F1	CGCGGATCCATGCCCAAGACGATCTATCGACCA	Cloning *srpA* into pTRG
srpA-H-F2	CGGAATTCCATGCCCAAGACGATCTATCGACCA	Cloning *srpA* into pBT
srpA-H-R	CCCTCGAGTCACTGCCGCGCTGTCGTG	
RNA-P-F	CTCATTCTCAAAGACCCG	Promoter fragments of the *gp058* gene for EMSA assay to determine the binding site

RNA-P-R	CCTCTTAATCAAGGATGTG	
BS-1-F	TATTGCAATTTCTTTGGCCG	
BS-2-F	CCATATGCTGGCGGTAT	
BS-4-F	TTCTTTGGCCGTGCCATA	
BS-5-F	GCGGTATCTCCAAATCGG	
BS-10-F	GGTATCTCCAAATCGGATATGC	
BS-21-F	GTATCTCCAAATCGGATATGC	
BS-20-R	GTCATTAGCCAGCATATCC	
BS-21-R	GGGGTCTAGGTCATTAGCC	
BS-23-R	ATATCCGATTTGGAGATACCGC	
BS-24-R	CCAGCATATCCGATTTGGAG	
BS-25-R	TATCCGATTTGGAGATACC	
BS-26-R	ATCCGATTTGGAGATACCG	
RNA-E-F	CGGGATCCGACAAGGGGAATGAGAGAA	The *gp058* gene with its own promoter for complement experiment
RNA-E-R	CGGGATCCACTGGAGGGGTAGGTCTTC	
srpA-F	CGCGGATCCATGCCCAAGACGATCTATCGACCA	Overexpression of *srpA* in pQE30
srpA-R	CCCAAGCTTTCACTGCCGCGCTGTCGTG	
DNAI-P-F	AGCGGTATTGTAGGGTCTG	The promoter region of gene *gp104* for EMSA assay
DNAI-P-R	CAGTCCGATTCCAGAGTCA	
DNAII-P-F	AACCTGATTAACGTGAAGC	The promoter region of gene *gp105* for EMSA assay
DNAII-P-R	TCCTCCTGTAAACGACGA	
PA1077-F	CGAGTGAGCCGTGTCCCC	The promoter region of gene *PA1077* for EMSA assay
PA1077-R	CGTCGCCAACACTTCAGCA	
PA1826-F	GAACTGGCGTAAGTCGGCC	The promoter region of gene *PA1826* for EMSA assay
PA1826-R	GCCAGGCCGATGCCC	
PA3766-F	CGCCTTTCTCTCCTTCAA	The promoter region of gene *PA3766* for EMSA assay
PA3766-R	CGGCGACTACGACTTTAC	
K5_gp053-F	AAGAGTCCGTTGGCGTAA	The promoter region of gene *gp053* for EMSA assay
K5_gp053-R	TCGCTTGTAGTATTCGTAGAC	
K5_gp125-F	CAACCACGAAGGCTAATC	The promoter region of gene *gp125* for EMSA assay
K5_gp125-R	GCCACAACGCTTTGAACA	
M13-47-cy3	CGCCAGGGTTTTCCCAGTCACGAC	Amplification of the promoter regions cloned in the T-vector pMD19 (simple)
RV-M-cy3	GAGCGGATAACAATTTCACACAGG	
HZW-P1	CGGAATTCCGCCGCCGCGAAATTTTC	The upstream fragment for knocking out the *srpA* gene
HZW-P2	CGGGATCCGATCGAGATAGCCCATCATT	
HZW-P3	CGGGATCCGGTCGGTAGCTACGTGGG	The downstream fragment for knocking out the *srpA* gene

HZW-P4	GGGGTACCGCCGACCACTACGCTCCT	
RNA-F	CCGAAGCTTGGTCCTCAAGACCTTAGCAT	The *gp058* gene driven by the P_{lac} promoter for complement experiment
RNA-R	CGCAAGCTTCATGAATCTATCGGCACTCC	

a: The underlined sequences stand for the recognition sites of the restriction enzymes.

b: The primers RNA-H-F1 and RNA-H-R are used for amplification of *gp058* into pTRG. The primers RNA-H-F2 and RNA-H-R are used for amplification of *gp058* into pBT.

c: The primers srpA-H-F1 and srpA-H-R are used for amplification of *srpA* into pTRG. The primers srpA-H-F2 and srpA-H-R are used for amplification of *srpA* into pBT.

d: The primers M13-47-cy3 and RV-M-cy3 are both labeled with the fluorescent dye cy3 at 5' ends. The amplicons are used as probes in the EMSA assay.

Supplementary References

1. Bradley TJ, Khan NH. The production of extracellular lipids by Pseudomonas Aeruginosa NCTC 2000 in stationary liquid media containing macrogols. *The Journal of pharmacy and pharmacology* **26**, 900-902 (1974).

2. Wang J, Mushegian A, Lory S, Jin S. Large-scale isolation of candidate virulence genes of Pseudomonas aeruginosa by in vivo selection. *Proceedings of the National Academy of Sciences of the United States of America* **93**, 10434-10439 (1996).

3. Cui X*, et al.* Characterization of Pseudomonas aeruginosa Phage C11 and Identification of Host Genes Required for Virion Maturation. *Scientific reports* **6**, 39130 (2016).

4. Masuda N, Ohya S. Cross-resistance to meropenem, cephems, and quinolones in Pseudomonas aeruginosa. *Antimicrobial agents and chemotherapy* **36**, 1847-1851 (1992).

5. Liberati NT*, et al.* An ordered, nonredundant library of Pseudomonas aeruginosa strain PA14 transposon insertion mutants. *Proceedings of the National Academy of Sciences of the United States of America* **103**, 2833-2838 (2006).

6. Brenner S. The genetics of Caenorhabditis elegans. *Genetics* **77**, 71-94 (1974).

7. Hoang TT, Karkhoff-Schweizer RR, Kutchma AJ, Schweizer HP. A broad-host-range Flp-FRT recombination system for site-specific excision of chromosomally-located DNA sequences: application for isolation of unmarked Pseudomonas aeruginosa mutants. *Gene* **212**, 77-86 (1998).

8. Totten PA, Lory S. Characterization of the type a flagellin gene from Pseudomonas aeruginosa PAK. *J Bacteriol* **172**, 7188-7199 (1990).

9. Schweizer HP. Escherichia-Pseudomonas shuttle vectors derived from pUC18/19. *Gene* **97**, 109-121 (1991).

10. West SE, Schweizer HP, Dall C, Sample AK, Runyen-Janecky LJ. Construction of improved Escherichia-Pseudomonas shuttle vectors derived from pUC18/19 and sequence of the region required for their replication in Pseudomonas aeruginosa. *Gene* **148**, 81-86 (1994).

11. Li M*, et al.* HigB of Pseudomonas aeruginosa Enhances Killing of Phagocytes by Up-Regulating the Type III Secretion System in Ciprofloxacin Induced Persister Cells. *Frontiers in cellular and infection microbiology* **6**, 125 (2016).

12. Li L, Pan X, Cui X, Sun Q, Yang X, Yang H. Characterization of Pseudomonas aeruginosa phage K5 genome and identification of its receptor related genes. *Journal of basic microbiology* **56**, 1344-1353 (2016).
