# Supplementary material for: Regulatory protein SrpA controls phage infection and core cellular processes in Pseudomonas aeruginosa
Source: Nat Commun. 2018 May 10;9:1846. doi: 10.1038/s41467-018-04232-6 (PMC5945682; doi:10.1038/s41467-018-04232-6)
Supplement: Supplementary file 3 — Description of Additional Supplementary Files [file 41467_2018_4232_MOESM3_ESM.pdf]

Description of Additional Supplementary Files

File Name: Supplementary Data 1
Description: **PAK_AR2A-VS-s1_9A. GeneDiffExp.** Transcriptome analysis of the strain PAK-AR2 and its derivative strain 1-9, which has the Tn*5G* transposon inserted in the *srpA* gene (Supplementary Table 2). The differentially expressed genes (DEGs) were determined between PAK-AR2 and 1-9 with the standards of false discovery rate (FDR) ≤ 0.001, fold change |log2Ratio|≥1. KEGG database was used to classify the genes with significant differential expression based on their functions. The genome of *P. aeruginosa* PAO1 (NC_002516.2) was used as reference for annotation.

File Name: Supplementary Data 2
Description: **SrpA-like protein sequences from the GenBank with E-value better than 5E-8.** The protein sequences of SrpA homologs in microorganisms were retrieved by searching Non-redundant protein sequences (nr) database using PSI-BLAST (Position-Specific Iterated BLAST) in NCBI. Total 1000 homologous sequences were selected with the E-value lower than the threshold of 5E-08.

File Name: Supplementary Data 3
Description: **SrpA-like proteins from *P. aeruginosa* and phages from the GenBank with the E-value better than 0.001.** The protein sequences of SrpA homologs in *P. aeruginosa* and phages were retrieved by searching Non-redundant protein sequences (nr) database using PSI-BLAST (Position-Specific Iterated BLAST) in NCBI. The homologous sequences were selected with E-value lower than the threshold of 0.001.

File Name: Supplementary Data 4
Description: **SrpA-like proteins from the genome database of *P. aeruginosa* with E value better than 9.2E-17.** The protein sequences of SrpA homologs in *P. aeruginosa* were retrieved by searching the genome database of *Pseudomonas*. The homologous sequences were selected with E-value lower than 9.2E-17.

File Name: Supplementary Data 5
Description: **16S rRNA gene sequences of 2156 strains from the database of *P. aeruginosa.*** The 16S rRNA gene sequences from *P. aeruginosa* strains were retrieved by searching the genome database of *Pseudomonas*. Only one 16S rRNA gene sequence of each strain was used in the construction of the phylogenetic tree. pMD19 (Simple) is the name of a linearized vector with a single 3'-terminal thymidine at both ends. The plasmid is provided by Clontech with catalogue # 3271.
